# PALM-IST: Pathway Assembly from Literature Mining - an Information Search Tool

**DOI:** 10.1038/srep10021

**Published:** 2015-05-19

**Authors:** Sapan Mandloi, Saikat Chakrabarti

**Affiliations:** 1 Structural Biology and Bioinformatics Division, CSIR-Indian Institute of Chemical Biology, Kolkata 700032, India

## Abstract

Manual curation of biomedical literature has become extremely tedious process due to its exponential growth in recent years. To extract meaningful information from such large and unstructured text, newer and more efficient mining tool is required. Here, we introduce PALM-IST, a computational platform that not only allows users to explore biomedical abstracts using keyword based text mining but also extracts biological entity (*e.g*., gene/protein, drug, disease, biological processes, cellular component, etc.) information from the extracted text and subsequently mines various databases to provide their comprehensive inter-relation (*e.g*., interaction, expression, etc.). PALM-IST constructs protein interaction network and pathway information data relevant to the text search using multiple data mining tools and assembles them to create a meta-interaction network. It also analyzes scientific collaboration by extraction and creation of “co-authorship network,” for a given search context. Hence, this useful combination of literature and data mining provided in PALM-IST can be used to extract novel protein-protein interaction (PPI), to generate meta-pathways and further to identify key crosstalk and bottleneck proteins. PALM-IST is available at www.hpppi.iicb.res.in/ctm.

Biomedical research has shifted from studying individual gene and protein to complete biological system. In today’s age of “big data biology” embraced with overwhelming amount of publication records and of high-throughput data, automated and efficient text and data mining platforms are absolutely essential to access and present biological information into more computable and comprehensible form^1^,^2^,^3^. Biomedical literature scan is the key to understand large amount of data generated in experiments and to retrieve novel information from them. Various search engines, for example PubMed^4^, MedlineRanker^5^, iPubMed^6^, GoPubMed^7^, XploreMed^8^, Whatizit^9^ and others have been successfully launched to retrieve meaningful information from text mining. ABNER^10^, ABGene^11^, LingPipe (Alias-i. 2008) are some of the entity recognition tools whose goal is to identify biological terms like gene name, drug name within biomedical literature. Similarly, multiple methods^12^,^13^,^14^,^15^,^16^,^17^,^18^,^19^,^20^,^21^,^22^,^23^ have been employed to extract meaningful information regarding interaction and inter-relation between bio-molecules (*e.g.,* gene/protein, drugs, etc.) and bio-processes (*e.g.,* cellular pathways and processes) using text and data mining approaches.

However, for meaningful knowledge discovery, more sophisticated amalgamation of literature analysis and biomedical data mining is required where text-mining solutions integrate with other data resources containing information regarding pathways, protein-protein interactions, gene expression and functional genomics. Intelligent combination of text and data mining might help researchers to discover hitherto undiscovered knowledge about novel protein-protein interaction information, gene-disease, and gene-drug relation. Scattered examples are available where information about gene-gene interactions^24^, alternative splicing^25^, functional analysis of mutations^26^, phosphorylation sites^27^, and regulatory sites^28^ were extracted using text mining based approaches. Protein-protein interaction (PPI), signaling, regulatory and metabolic pathways^29^,^30^,^31^ are becoming increasingly important part of computational systems biology and it is crucial to merge these various interactions of biological entities for better understanding of a biological system or context (*e.g*., diseases). Hence, implementation of an accurate, robust and automated platform aiming to integrate mining, interaction and pathway data towards development of meta-interaction networks is due.

Here, we present PALM-IST, which aims to provide intelligent combination of literature analysis and biomedical data mining. In PALM-IST, a user not only receives more relevant text results for various combination keywords but also can access important information regarding genes/proteins, gene expression, interactions, biological pathways, drug-disease association that are deemed to be connected to the search result. To the best of our knowledge, PALM-IST is the only platform where text mining, database retrieval (data mining), and pathway assembly have been done simultaneously. PALM-IST constructs and assembles protein network and pathway information data relevant to the search using multiple data mining tools. Biological pathways (signaling and/or metabolic) of the observed proteins and their PPI network are overlaid to provide a meta-interaction network of cellular systems. Such assemblies of pathways can prove to be crucial for generation of novel meta-pathways containing key crosstalk and bottleneck proteins. PALM-IST also emphasized on scientific collaboration in the form of “co-authorship network,” in which nodes represent author name and edges represent co-authorship connection. Co-authorship networks are useful and can tell interesting features of academic communities. Thus, PALM-IST can become an important platform to aid large scale system biology based research where multiple genes/proteins and pathways are required to be examined to simultaneously for better understanding of the cellular complexity.

## Results

### Input and output features of PALM-IST server

#### Input option

A two tier keyword (primary and secondary) based search engine is introduced in PALM-IST server. Topic (*e.g*., glioblastoma or haemophilia) and author (*e.g*., Weinberg RA) based searches can be performed simultaneously and/or separately. All primary keywords along with their synonyms and acronyms are searched using AND (specified as “,” symbol) or OR (specified as “|” symbol) Boolean gates in NCBI PubMed^4^ using NCBI Eutils. Abstracts retrieved from primary keyword based search are further sorted for all possible combinations of secondary keywords (separated by new line) along with their synonyms. Option of exclusion of certain keywords can also be invoked in PALM-IST input option.

#### Output option

**Figure 1:**
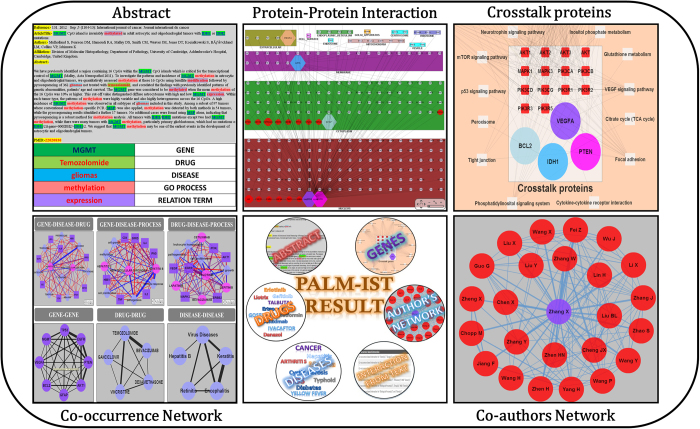
Snapshots of PALM-IST output options with example primary and secondary keywords.

The output of the PALM-IST server can be subdivided into five parts. Following section briefly describes each of the output using an example set of keywords (primary and secondary) based search. Fig. 1 provides a general overview of the output options of the PALM-IST server. All the output numbers provided in this manuscript are derived from PUBMED search performed in the month of December 2014.

#### Abstract result

Abstracts retrieved for primary keyword based search are displayed and highlighted with the bio-entity words (gene/protein, drugs, and diseases and biological processes) and relation terms (*e.g*., modulate, elevate, etc.). PALM-IST provides a unique option of simultaneous literature mining using multiple secondary keywords where abstracts are sorted for all possible combination of secondary query words. 63992 abstract containing articles are retrieved using an example literature search with primary keywords “*Glioblastoma|Glioma|Brain tumor|Brain cancer*” (“|” denotes OR gate) and secondary keywords like “*EGFR<new line>TP53<new line>Erlotinib|Gefitinib*”. Results for all single secondary keywords and in combination are mutually exclusive. Synonyms of genes for secondary keywords (*e.g*., p53, TP53, tumor protein p53) are automatically used in abstract search. For example, out of 63992 abstracts only 2 abstracts were found where all the three secondary keywords or their synonyms are present. However, this could easily be done using advance keywords search in PubMed, but to retrieve abstracts for the other combinations, such as EGFR and Erlotinib|Gefitinib or TP53 and EGFR, it would require separate PubMed searches. In PALM-IST abstracts for all the combinations can be retrieved in single search. This becomes really useful option when a large number of secondary keywords are required to be searched. Result for secondary keywords can be used to write the summary of abstract, which in principle can be utilized for refined and curated text search.

#### Gene result

This section of the PALM-IST server deals with entity recognition of genes/proteins and tagging of abstracts containing the gene/protein names or their synonyms. It provides a list of human genes/proteins that are frequently found in the abstracts yielded by the primary and secondary keywords based search. Protein-protein interaction (PPIs) with two tiers of interaction (1^st^ level and 2^nd^ level interacting protein) for each observed protein is displayed with subcellular compartmentalization. Biological pathways with which each observed protein is involved are shown in a network display where crosstalk proteins (proteins that connect multiple pathways) are identified and emphasized. Biological pathways (signaling and/or metabolic) of the observed proteins and their PPI network are overlaid to provide a meta-interaction network of cellular systems. Secondary information including gene summary, gene loci, Swiss-Prot/Ensembl code, three dimensional structure, and single nucleotide polymorphism (SNP) for each observed gene are provided on mouse click. Molecular expression data of the listed genes are provided where users can find up- and down-regulation patterns of those genes in numerous experimental conditions^32^. Molecular expression profile of identified gene/proteins is also overlaid onto the assembled pathway network using user defined expression context and datasets. Figure S1 provides an example of expression mapping onto the p53 signaling pathway overlaid with protein-protein interaction information.

In this section, options are also provided for the users to merge PPIs and pathways of multiple (maximum 5 proteins) proteins to create a meta-interaction network. This broadens the scope of visualizing large interaction and pathway information data within a single visualization window. Co-occurrence based connections between multiple genes can be extracted within this section of the PALM-IST server. Authors and co-authorship networks extracted from the publications in which the particular protein and the primary keywords co-occur is also generated.

For example, 9923 genes were found from 63992 PubMed abstracts obtained with *Glioblastoma* OR *Glioma* OR *Brain tumor* OR *Brain cancer* as primary keywords. Top 10 most frequently found genes/proteins within these abstracts are TP53 (tumor protein p53; 1559 abstracts), EGFR (epidermal growth factor receptor; 1520 abstracts), GFAP (glial fibrillary acidic protein; 1180 abstracts), AKT1 (v-akt murine thymoma viral oncogene homolog 1; 964 abstracts), VEGF (vascular endothelial growth factor; 859 abstracts), MGMT (O-6-methylguanine-DNA methyltransferase; 824 abstracts), PTEN (phosphatase and tensin homolog; 622 abstracts), BCL2 (B-cell CLL/lymphoma 2; 507 abstracts), EGF (epidermal growth factor; 476 abstracts) and TNF(tumor necrosis factor; 475 abstracts). All together 1272 unique protein-protein interactions including 332 interacting proteins were extracted for these 10 proteins. Merging of pathways involving these 10 proteins yielded 73 unique pathways and 1366 crosstalk proteins that connect at least two pathways. Mitogen-activated protein kinase 3 and 1 (MAPK3 and MAPK1), phosphatidylinositol-4,5-bisphosphate 3-kinase (PIK3 family), AKT1 (v-akt murine thymoma viral oncogene homolog 1), mitogen-activated protein kinase kinase 1 (MAP2K1) and RAS p21 protein activator (RAS) turn out to be the top 5 enzymes that crosslink maximum numbers of biological pathways (41, 37, 34, 31 and 30 pathways, respectively).

#### Drugs and Disease result

Abstracts yielded by primary and secondary keywords based search are scanned and sorted based on the presence of 925 approved drugs and 3813 disease names. Biological pathways (signaling and/or metabolic) related to these drugs and diseases are presented. Similar to the gene/protein section, co-occurrence based connections between multiple drugs and diseases can be extracted within this section of the PALM-IST server. Names of the experts or authors who are frequently publishing scientific papers related to the drugs and diseases observed within the searched abstracts are presented in tabular and network display.

#### Co-occurrence based interaction result

In this section PALM-IST offers text based co-occurrence of genes, drugs, diseases, and biological processes. Triad combinations of gene-drugs-disease and gene-disease-process are extracted and most frequent combinations are provided in tabular and network display option. For example, for the abstracts obtained with *Glioblastoma* OR *Glioma* OR *Brain tumor* OR *Brain cancer* as primary keywords, most frequent gene-drug-disease triads are found to be MGMT-Temozolomide-Glioma (534 abstracts), VEGF-Bevacizumab-Glioma (156 abstracts), EGFR-Erlotinib-Glioma (99 abstracts), etc. Similarly, most frequent gene-disease-process triad combinations are EGFR-Glioma|Glioblastoma-Growth (1301 abstracts), VEGFA-Glioma|Glioblastoma-Growth (800 abstracts), MGMT-Glioma|Glioblastoma-Methylation (609 abstracts), AKT1-Glioma|Glioblastoma-Signaling (598 abstracts), VEGFA-Neoplasms-Angiogenesis (377 abstracts), etc. In addition to the triads, various combinations of pairs of genes, drugs, diseases, and biological processes are also extracted from the searched abstracts based on their co-occurrence. The observation of MGMT (O-6-methylguanine-DNA methyltransferase), Temozolomide and methylation as the most frequently observed (515 abstracts) gene-drug-biological process triad is in fact quite fascinating and can act as a proof of concept for the discovery of new knowledge of association between genes, drugs, diseases, and biological processes. The strong co-occurrence of MGMT, Temozolomide and methylation extracted by PALM-IST clearly indicates crucial association of them with Glioma. This is indeed the case as some tumors become sensitive to Temozolamide, via epigenetic silencing of MGMT/AGT gene^33^. Similarly, brain tumors with MGMT protein show little responce to Temozolomide^34^.

#### Author’s network results

Author’s statistics and network is an interesting feature of PALM-IST server. It provides detailed countrywide publication statistics represented in tabular and interactive global map format. Similarly, most frequent authors and their co-authoring relationship for a given literature search are provided in network based display using Cytoscape Web^35^ applet. For the abstracts obtained with *Glioblastoma* OR *Glioma* OR *Brain tumor* OR *Brain cancer* as primary keywords, most papers are published from United States Of America (20945 papers) while most frequent authors and co-authors are Darell D Bigner from Duke University and Henry S Friedman from Duke University Neurosurgery Division who are renowned experts of the Glioma field for the last few decades. These author and co-author’s networks not only provide an idea about the experts of the fields but are also quite useful in revealing many interesting features of academic communities^36^ and are helpful in generating new and valuable information relevant to the strategic planning, implementation and monitoring of scientific policies and programs^37^,^38^. Disambiguation of author’s name is an important but challenging pre-processing step in literature mining. However, it is out of the scope of this paper to pre-process and disambiguate all the authors’ name. We have used author’s name and initials provided by the PubMed^4^.

#### PALM-IST statistics for multiple types of diseases as query keywords

Other than the above mentioned example primary keyword (*i.e*., “*Glioblastoma|Glioma|Brain tumor|Brain cancer*”), various other disease names were used as query keywords. These diseases were grouped into four categories: a) metabolic disease, b) cancer, c) infectious disease and d) other diseases. Table S1 outlines total number of abstracts, gene/protein, drugs, PPI, pathways, crosstalk protein, signaling-metabolic common proteins, and co-authors statistics extracted for these diseases when used as query keywords in the PALM-IST server.

#### PALM-IST report

In addition to the web-based interactive display, a summarized report containing associated genes, drugs, disease and authors is generated and sent through email on user’s request for their respective input query. This report contains number and list of the extracted abstracts, protein-protein interaction, cross-talk proteins, frequent authors, associated drugs, diseases, pathways and genes/proteins for a given keyword search.

#### Validation and Benchmark

Table 1 outlines a qualitative comparison highlighting various features of the PALM-IST server with respect to other freely available tools.

We validated the performance of the bio-entity recognition component of PALM-IST using various gold standard corpuses^39^ (GSC) such as BioCreative corpus^39^, NCBI Disease corpus^40^, CHEMDNer corpus^41^ (BioCreative task IV), Arizona disease corpus (AZDC)^42^ etc. Table 2 provides the performance measures for the bio-entity validations (see Methods and supplementary file 1 for details). Programs shaded in grey in Table 2 are used in the PALM-IST.

Performance of gene name recognition component of the PALM-IST server aided by the GeneTUKIT^43^ was compared with that of two other programs namely BANNER^44^ and Abgene^11^ using the standard BioCreative task II^45^ gene mention corpus. F-measure of the PALM-IST gene name recognition component calculated from the precision and recall values was found to be higher than those of the two above mentioned programs. Similarly, gene normalization component of the PALM-IST server aided by the GenNorm^46^ software was benchmarked against the BioCreative task III^47^ and task II^48^ corpuses. Performances of the PALM-IST gene normalization component were observed to be higher than GNAT^49^ and Moara^50^ when compared for all species (BioCreative task III) and for human (BioCreative task II) gene normalization, respectively. However, it must be noted that for human gene normalization, GNAT^49^ outperforms the PALM-IST component.

Disease name recognition aided by the DNorm^51^ software was benchmarked against the NCBI and Arizona disease corpuses^40^,^42^. In both cases PALM-IST embedded component (*i.e*., DNorm) outperformed the MetaMap^52^ package. Similarly, Pubtator^53^ based Chemical/Drug name recognition component also provides better performance than that achieved by the Whatizit^9^ package (Table 2).

The Comparative Toxicogenomics Database^54^ (CTD) includes curated data describing association between genes/drugs/pathway and various environmentally influenced diseases. Here, we have validated the accuracy of genes/drugs/pathway associations suggested by PALM-IST text and data mining components using the CTD enlisted disease MESH terms as query keywords. The top 10 and 20 genes/drugs/pathways based on occurrence for each disease keyword search was compared against CTD enlisted associations. Table 3 provides the percentage of identical gene/drugs/pathway yielded by the PALM-IST keywords search based association and the CTD enlisted disease- gene/drugs/pathway association.

The co-authorship network and network derived features were verified against published networks provided in the PubNet^55^ server. PubNet co-author networks were re-created by the PALM-IST using the same query keywords/author based searches (Figure S2). Further, the networks and their features were compared (Table S2 in supplementary file 1) to show their similarities, which indirectly provide reliability of the PALM-IST co-author networks.

## Conclusion

Biomedical literature scan is critical to understand large amount of data generated in experiments and to retrieve novel information from them. PALM-IST constructs and assembles protein network and pathway information data relevant to the gene/proteins frequently observed within the searched text. Hence, PALM-IST can become an important platform to aid large scale system biology based research where multiple genes/proteins and pathways are required to be examined simultaneously for better understanding of the cellular complexity. A key challenge in cell biology is to understand the interconnectivity between its biochemical pathways with respect to extracellular signals. Hence, the assembled/interconnected network (supra-network) constructed via PALM-IST applications can help in generating new hypotheses and can discover emergent properties of the biological systems.

## Methods

### Methodology and Architecture of the server

**Figure 2:**
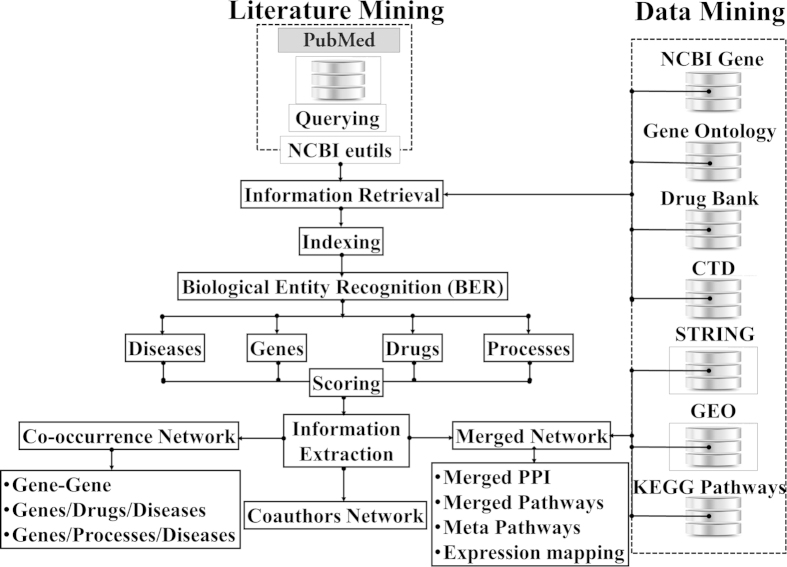
A schematic representation of the workflow of the PALM-IST methodology and architecture.

PALM-IST is developed on CGI-PERL based web architecture. Figure 2 shows a schematic representation of the workflow of the PALM-IST methodology and architecture. Following section briefly describes various features of the server.

### Input/Query

Multiple keywords of varied nature, such as genes, disease, drug, author names or any other word(s) can be provided as primary keyword input. Similarly, secondary keywords can also be mined in all possible combinations on abstracts retrieved from the primary keyword based search.

### Collection of bio-entity information

Information regarding genes, diseases, drugs, pathways, interactions, and expression data were collected from various well-established resources (complete list of resources and MYSQL indexed table size with relevant details are provided in Table S3 and S4 of supplementary file 1) are utilized within the PALM-IST server. Information regarding 15.5 million gene and 11 million taxonomic entries were collected from the NCBI resources and were further processed for indexing. Indexed genes/proteins were mapped onto 1.23 million cellular pathways collected from the Kyoto Encyclopedia of Genes and Genomes^29^ (KEGG) database and almost 40,000 Gene Ontologies collected from the GO database^56^. Protein-protein interaction information was collected from the STRING database^57^ and additional information regarding the 23184 human genes was extracted from the Genecards^58^ resources. Gene expression data was collected from the Gene Expression Omnibus^59^ (GEO) while drug-gene/drug-disease association information was extracted from the Comparative Toxicogenomics Database^54^ (CTD) and the DrugBank^60^ database.

### Indexing and Scoring

Till November 2014, 14361661 PubMed abstracts were indexed and processed in PALM-IST. Newer abstracts are downloaded and added on regular interval. Hypergeometric test was used to estimate the likelihood of the observation of a bio-entity by chance within a given text^61^. Following section briefly describes the hypergeometric and co-occurrence scoring between two bio-entities.

Hypergeometric test was used to estimate the likelihood of the observation of a bio-entity by chance within a given text. Gene/protein, drug, disease observed for a given text search are ranked based on the number of publications retrieved with the gene/drug/disease among the total number of publications linked to the gene/drug/disease. Hypergeometric test and score for the bio-entity was calculated using the contingency table (Table 4) and the following formula:


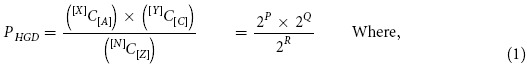



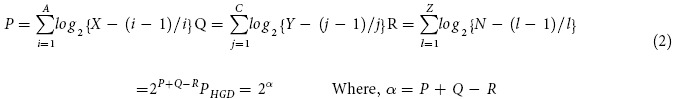



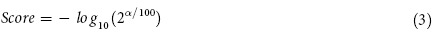


For a given primary query term(s) (*e.g*., Glioblastoma or Glioma) based text search, A is the number of publications that involve an observed bio-entity (*e.g*., TP53 or Gefitinib) where B is the number of publications that do not contain that particular bio-entity term. Similarly, C is the publications containing the bio-entity term (*e.g*., TP53) but not the query term(s) while D does not contain the particular bio-entity (*e.g*., TP53) and the query term(s), but contain other bio-entity (*e.g*., proteins) name. Y denotes the number of publication in which at least one bio-entity term is found (for example, articles containing at least one gene or one drug) but not the query term(s). ^X^*C*_A_, ^Y^*C*_C_, and ^N^*C*_Z_ [can also be represented as equation Eq. (2)] are the various combinations of publication with or without the presence of a particular bio-entity (*e.g*., TP53 or Gefitinib). *P*_*HGD*_ equation Eq. (1) is the probability of observing TP53 or Gefitinib for A or query-relevant publications by chance where *Score* equation Eq. (3) is the log transformation of the probability. As the combinations may result in very large numbers leading to very low probability values, we have utilized a *log*_*2*_ conversion followed by a division with a constant number (100). Higher the *Score* better is the significance of association between the bio-entity and the query term(s).

Score in Eq. (4) for co-occurrence based relation between two entities is calculated using the mutual information^62^ (MI). MI relates the joint probability of two items occurring [p(X,Y)] with respect to the probability of independent occurrence [p(X) . p(Y)]. The higher the MI value, the greater is the confidence in hypothesizing the co-occurrence.


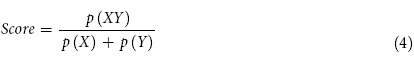



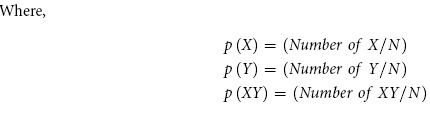
Where, N is the number of abstracts in query result (for query context) or complete PubMed database (for global context).

### Named Entity Recognition (NER)

Biological entity recognition (BER) is a part of named entity recognition (NER) where textual data is mined to identify^63^,^64^,^65^ relevant biological entities (*e.g*., genes, proteins, drugs, diseases, etc to facilitate their functional classification^66^). In PALM-IST we have used two open sourced, widely used programs GeneTUKit^43^ and GenNorm^46^ for gene name recognition and normalization, respectively. Similarly, DNorm^51^ was used for disease recognition and a dictionary-based lookup approach implemented in Pubtator^53^ was utilized for chemical/drug name recognition. Short descriptions about the methodology of these programs are provided in supplementary file 1.

### Information Extraction (IE)

PALM-IST extracts relations based on co-occurrence of bio-entities and presents in tabular and interactive network visualization manner aiding to understand the relationship between gene-gene, protein-protein, gene-disease-drug, disease-drug, gene-drug, gene-processes-disease and gene-processes. Indexing of association table is performed based on common publication containing two/three bio-entities. These associations as solely based on co-occurrence at abstract level. This approach is based on the assumption that co-occurrence of multiple biomedical concept in the same abstract is an indication of a functional link between those bio-entities.

### Network construction and visualization

Protein-protein interaction (PPI) data were collected from the STRING^57^ database. For each protein up to two-level interaction (interactors of direct interactor) were considered. The interactions were further divided into two classes, high (> = 0.7) and medium confidence (> = 0.4) based on the STRING confidence score. Each protein is tagged with its subcellular localization and the protein-protein interaction networks (PPIN) are displayed with subcellular compartmentalization to aid the visualization of interaction (Figure S3). PALM-IST combines biological pathway information with protein-protein interaction data by overlaying the pathway (directed) with PPI (undirected) network using Cytoscape web^35^ application. Similarly, PPIs and biological pathways associated with multiple proteins can be merged and assembled in PALM-IST. Molecular expression profile of the identified gene/proteins can also be overlaid onto the assembled pathway network using user defined expression context and datasets. Details regarding the workflow of expression mapping onto pathways and differential expression calculation can be found in the supplementary file 1 (Figure S4). In addition to these pathway assembly features, co-occurrence network of multiple genes, drugs, diseases and biological processes can be visualized in PALM-IST server via the Cytoscape web^35^ application. Associations between pairs of gene, drug, disease and process are identified based on their co-occurrence in the abstract. Two types of pair wise co-occurrence scores are calculated. Query context score and global context score, where query context score is meant to depict the significance of the co-occurrence within the abstract containing query term whereas global score signifies with respect to complete database size (see *Indexing and Scoring* section for details). Triad combinations of gene-drugs-disease and gene-disease-process are extracted based on abstracts with three bio-entities and most frequent combinations are provided in tabular and network display option. Triad combination score is calculated based on hypergeometric test (see *Indexing and Scoring* section for details). Network of co-occurrence can be visualized using Cytoscape web display where, nodes are bio-entities and edge represents abstracts connecting the corresponding bio-entities. Bio-entities are color coded according to their types and edge width is set on the basis of number of abstracts in which those bio-entities are co-occurred.

### Author’s statistics and co-authorship network

MySql based indexing is used to extract country statistics, authors and co-authors name. Google maps API is used to point country wise publication information on world map. Further, most frequent authors for a given text search is also extracted and co-authorship networks of the most frequent authors are provided within network based display using Cytoscape web^35^ applet.

### Evaluation of named entity recognition (NER)

BioCreative task II^45^ gene mention (BC2GM) corpus is concerned with the named entity extraction of gene and gene product mentioned in text. BC2GM test set containing 5000 sentences were utilized for gene mention programs’ evaluation. BioCreative task II^48^ gene normalization (BC2GN) is meant to link genes or gene products mentioned in the literature to standard database identifiers. BC2GN test set containing 252 articles were utilized for gene normalization programs’ evaluation. BioCreative task III^47^ gene normalization (BC3GN) containing 50 gold standard articles is meant to link gene or gene products mentions in full text literature. NCBI disease corpus^40^ is fully annotated at the mention and concept level to serve as a research resource for the biomedical natural language processing community. Test set contains 100 articles, which were utilized for disease normalization programs’ evaluation. AZDC corpus^42^ containing 2856 PubMed abstracts annotated for disease names (including symptoms etc.) and mapped to Concept Unique Identifiers (CUIs) of Unified Modeling Language System (UMLS), which were further utilized for evaluation. CHEMDNer Corpus^41^ provides detection of mentions of chemical compounds and drugs with the opportunity to compare the methods for chemical named entity recognition (NER) and indexing in a controlled environment. Test set contains 2478 articles, which were utilized for Chemical/Drug NER evaluation. Approved drug corpus was created by extracting the approved drugs (collected from DrugBank^60^) from the CHEMDNer corpus contains 982 articles and 1743 drug mention.

Performance of the algorithms/programs was evaluated by calculating the Recall, Precision and F-measure using the following formulae


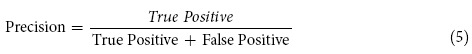



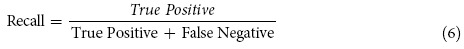



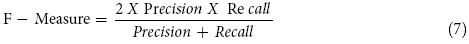


True and false positives are the number of bio-entities that were identified correctly and incorrectly, respectively.

## Author Contributions

S. M. collected and organized the data, developed the server and drafted the manuscript. S. C. drafted the manuscript and coordinated the project. All authors read and approved the final manuscript.

## Additional Information

**How to cite this article**: Mandloi, S. and Chakrabarti, S. PALM-IST: Pathway Assembly from Literature Mining - an Information Search Tool. *Sci. Rep.* **5**, 10021; doi: 10.1038/srep10021 (2015).

## Supplementary Material

Supporting Information Supplementary File 1

## Figures and Tables

**Table 1:** Qualitative comparison of the server/database features.

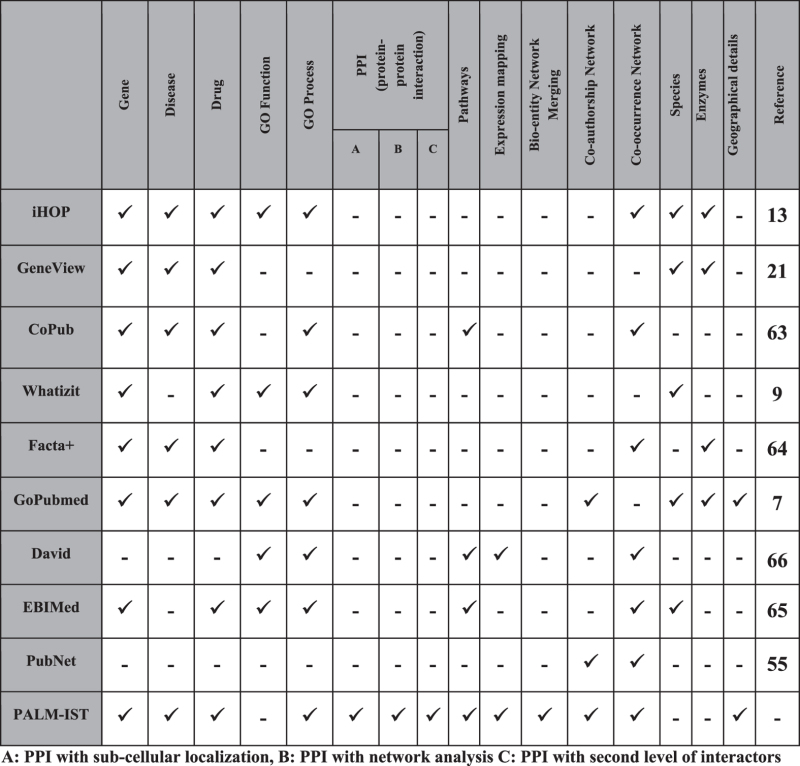

**Table 2:** Performance measures for bio-entity recognition.

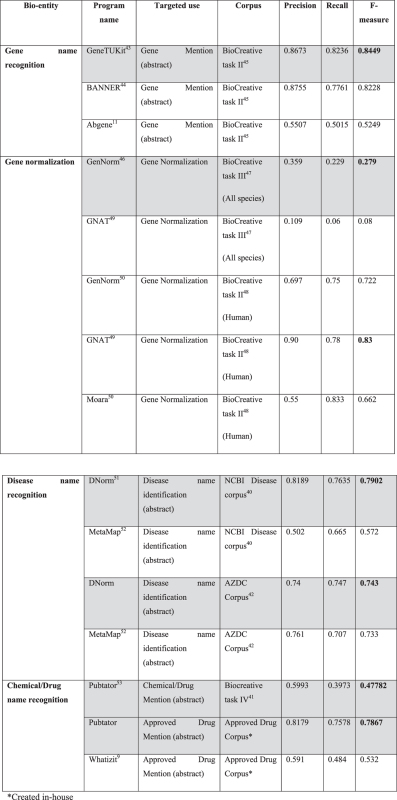

**Table 3:** Validation of gene/drug/pathway association with CTD enlisted diseases.

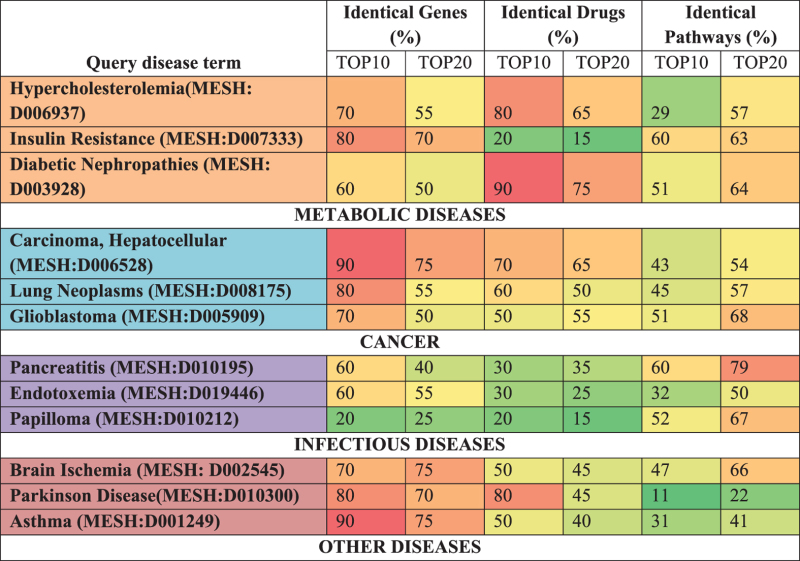

**Table 4:** Bio-entity contingency table.

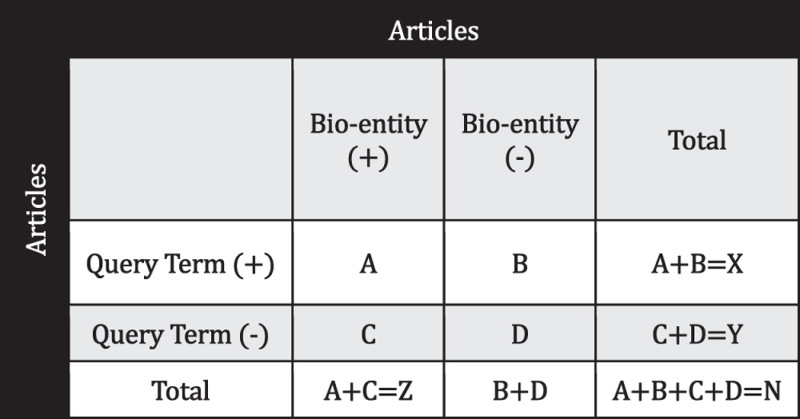

## References

[b1] Ananiadou S., Pyysalo S., Tsujii J. & Kell D. B. Event extraction for systems biology by text mining the literature. Trends Biotechnol. 28, 381–390, doi:10.1016/j.tibtech.2010.04.005 (2010). 20570001

[b2] Ananiadou S., Kell D. B. & Tsujii J. Text mining and its potential applications in systems biology. Trends Biotechnol. 24, 571–579, doi:10.1016/j.tibtech.2006.10.002 (2006). 17045684

[b3] Rebholz-Schuhmann D., Oellrich A. & Hoehndorf R. Text-mining solutions for biomedical research: enabling integrative biology. Nat. Rev. Genet. 13, 829–839, doi:10.1038/nrg3337 (2012). 23150036

[b4] Database resources of the National Center for Biotechnology Information. Nucleic Acids Res. 42, D7–17, doi:10.1093/nar/gkt1146 (2014). 24259429 PMC3965057

[b5] Fontaine J. F. *et al.* MedlineRanker: flexible ranking of biomedical literature. Nucleic Acids Res. 37, W141–146, doi:10.1093/nar/gkp353 (2009). 19429696 PMC2703945

[b6] Wang J. *et al.* Interactive and fuzzy search: a dynamic way to explore MEDLINE. Bioinformatics 26, 2321–2327, doi:10.1093/bioinformatics/btq414 (2010). 20624778

[b7] Doms A. & Schroeder M. GoPubMed: exploring PubMed with the Gene Ontology. Nucleic Acids Res. 33, W783–786, doi:10.1093/nar/gki470 (2005). 15980585 PMC1160231

[b8] Perez-Iratxeta C., Perez A. J., Bork P. & Andrade M. A. Update on XplorMed: A web server for exploring scientific literature. Nucleic Acids Res. 31, 3866–3868 (2003). 12824439 10.1093/nar/gkg538 PMC168945

[b9] Rebholz-Schuhmann D., Arregui M., Gaudan S., Kirsch H. & Jimeno A. Text processing through Web services: calling Whatizit. Bioinformatics 24, 296–298, doi:10.1093/bioinformatics/btm557 (2008). 18006544

[b10] Settles B. ABNER: an open source tool for automatically tagging genes, proteins and other entity names in text. Bioinformatics 21, 3191–3192, doi:10.1093/bioinformatics/bti475 (2005). 15860559

[b11] Tanabe L. & Wilbur W. J. Tagging gene and protein names in biomedical text. Bioinformatics 18, 1124–1132 (2002). 12176836 10.1093/bioinformatics/18.8.1124

[b12] Chen H. & Sharp B. M. Content-rich biological network constructed by mining PubMed abstracts. BMC Bioinformatics 5, 147, doi:10.1186/1471-2105-5-147 (2004). 15473905 PMC528731

[b13] Hoffmann R. & Valencia A. Implementing the iHOP concept for navigation of biomedical literature. Bioinformatics 21 **Suppl 2**, ii252–258, doi:10.1093/bioinformatics/bti1142 (2005). 16204114

[b14] Cheng D. *et al.* PolySearch: a web-based text mining system for extracting relationships between human diseases, genes, mutations, drugs and metabolites. Nucleic Acids Res. 36, W399–405, doi:10.1093/nar/gkn296 (2008). 18487273 PMC2447794

[b15] Corney D. P., Buxton B. F., Langdon W. B. & Jones D. T. BioRAT: extracting biological information from full-length papers. Bioinformatics 20, 3206–3213, doi:10.1093/bioinformatics/bth386 (2004). 15231534

[b16] He M., Wang Y. & Li W. PPI finder: a mining tool for human protein-protein interactions. PLoS One 4, e4554, doi:10.1371/journal.pone.0004554 (2009). 19234603 PMC2641004

[b17] Kim S. *et al.* PIE: an online prediction system for protein-protein interactions from text. Nucleic Acids Res. 36, W411–415, doi:10.1093/nar/gkn281 (2008). 18508809 PMC2447724

[b18] Kemper B. *et al.* PathText: a text mining integrator for biological pathway visualizations. Bioinformatics 26, i374–381, doi:10.1093/bioinformatics/btq221 (2010). 20529930 PMC2881405

[b19] Nikitin A., Egorov S., Daraselia N. & Mazo I. Pathway studio--the analysis and navigation of molecular networks. Bioinformatics 19, 2155–2157 (2003). 14594725 10.1093/bioinformatics/btg290

[b20] Tsuruoka Y., Miwa M., Hamamoto K., Tsujii J. & Ananiadou S. Discovering and visualizing indirect associations between biomedical concepts. Bioinformatics 27, i111–119, doi:10.1093/bioinformatics/btr214 (2011). 21685059 PMC3117364

[b21] Thomas P., Starlinger J., Vowinkel A., Arzt S. & Leser U. GeneView: a comprehensive semantic search engine for PubMed. Nucleic Acids Res. 40, W585–591, doi:10.1093/nar/gks563 (2012). 22693219 PMC3394277

[b22] Fang Y. C., Lai P. T., Dai H. J. & Hsu W. L. MeInfoText 2.0: gene methylation and cancer relation extraction from biomedical literature. BMC Bioinformatics 12, 471, doi:10.1186/1471-2105-12-471 (2011). 22168213 PMC3266364

[b23] Rzhetsky A. *et al.* GeneWays: a system for extracting, analyzing, visualizing, and integrating molecular pathway data. J. Biomed. Inform. 37, 43–53, doi:10.1016/j.jbi.2003.10.001 (2004). 15016385

[b24] Hoffmann R. & Valencia A. A gene network for navigating the literature. Nat. Genet. 36, 664, doi:10.1038/ng0704-664 (2004). 15226743

[b25] Shah P. K., Jensen L. J., Boue S. & Bork P. Extraction of transcript diversity from scientific literature. PLoS Comput. Biol. 1, e10, doi:10.1371/journal.pcbi.0010010 (2005). 16103899 PMC1183516

[b26] Horn F., Lau A. L. & Cohen F. E. Automated extraction of mutation data from the literature: application of MuteXt to G protein-coupled receptors and nuclear hormone receptors. Bioinformatics 20, 557–568, doi:10.1093/bioinformatics/btg449 (2004). 14990452

[b27] Hu Z. Z., Narayanaswamy M., Ravikumar K. E., Vijay-Shanker K. & Wu C. H. Literature mining and database annotation of protein phosphorylation using a rule-based system. Bioinformatics 21, 2759–2765, doi:10.1093/bioinformatics/bti390 (2005). 15814565

[b28] Saric J., Jensen L. J., Ouzounova R., Rojas I. & Bork P. Extraction of regulatory gene/protein networks from Medline. Bioinformatics 22, 645–650, doi:10.1093/bioinformatics/bti597 (2006). 16046493

[b29] Kanehisa M. & Goto S. KEGG: kyoto encyclopedia of genes and genomes. Nucleic Acids Res. 28, 27–30 (2000). 10592173 10.1093/nar/28.1.27 PMC102409

[b30] Vastrik I. *et al.* Reactome: a knowledge base of biologic pathways and processes. Genome Biol. 8, R39, doi:10.1186/gb-2007-8-3-r39 (2007). 17367534 PMC1868929

[b31] Fazekas D. *et al.* SignaLink 2 - a signaling pathway resource with multi-layered regulatory networks. BMC Syst. Biol. 7, 7, doi:10.1186/1752-0509-7-7 (2013). 23331499 PMC3599410

[b32] Petryszak R. *et al.* Expression Atlas update--a database of gene and transcript expression from microarray- and sequencing-based functional genomics experiments. Nucleic Acids Res. 42, D926–932, doi:10.1093/nar/gkt1270 (2014). 24304889 PMC3964963

[b33] Stupp R. *et al.* Effects of radiotherapy with concomitant and adjuvant temozolomide versus radiotherapy alone on survival in glioblastoma in a randomised phase III study: 5-year analysis of the EORTC-NCIC trial. Lancet. Oncol. 10, 459–466, doi:10.1016/S1470-2045(09)70025-7 (2009). 19269895

[b34] Hegi M. E. *et al.* MGMT gene silencing and benefit from temozolomide in glioblastoma. N. Engl. J. Med. 352, 997–1003, doi:10.1056/NEJMoa043331 (2005). 15758010

[b35] Lopes C. T. *et al.* Cytoscape Web: an interactive web-based network browser. Bioinformatics 26, 2347–2348, doi:10.1093/bioinformatics/btq430 (2010). 20656902 PMC2935447

[b36] Newman M. E. Coauthorship networks and patterns of scientific collaboration. Proc. Natl. Acad. Sci. U S A 101 **Suppl 1**, 5200–5205, doi:10.1073/pnas.0307545100 (2004). 14745042 PMC387296

[b37] Morel C. M., Serruya S. J., Penna G. O. & Guimaraes R. Co-authorship network analysis: a powerful tool for strategic planning of research, development and capacity building programs on neglected diseases. PLoS Negl. Trop. Dis. 3, e501, doi:10.1371/journal.pntd.0000501 (2009). 19688044 PMC2721762

[b38] Catala-Lopez F. *et al.* Coauthorship and institutional collaborations on cost-effectiveness analyses: a systematic network analysis. PLoS One 7, e38012, doi:10.1371/journal.pone.0038012 (2012). 22666435 PMC3362536

[b39] Blaschke C., Hirschman L., Yeh A. & Valencia A. Critical assessment of information extraction systems in biology. Comp. Funct. Genomics 4, 674–677, doi:10.1002/cfg.337 (2003). 18629031 PMC2447314

[b40] Dogan R. I., Leaman R. & Lu Z. NCBI disease corpus: a resource for disease name recognition and concept normalization. J. Biomed. Inform. 47, 1–10, doi:10.1016/j.jbi.2013.12.006 (2014). 24393765 PMC3951655

[b41] Arighi C. N. *et al.* BioCreative-IV virtual issue. Database (Oxford) 2014, doi:10.1093/database/bau039 (2014). PMC4030502 24852177

[b42] Leaman R. *et al.* Enabling Recognition of Diseases in Biomedical Text with Machine Learning : Corpus and Benchmark. Proceedings of the 3rd International Symposium on Languages in Biology and Medicine , 82–89 (2009).

[b43] Huang M., Liu J. & Zhu X. GeneTUKit: a software for document-level gene normalization. Bioinformatics 27, 1032–1033, doi:10.1093/bioinformatics/btr042 (2011). 21303863 PMC3065680

[b44] Leaman R. & Gonzalez G. BANNER: an executable survey of advances in biomedical named entity recognition. Pac. Symp. Biocomput. , 652–663, doi:10.1142/9789812776136_0062 (2008). 18229723

[b45] Smith L. *et al.* Overview of BioCreative II gene mention recognition. Genome Biol. 9 **Suppl 2**, S2, doi:10.1186/gb-2008-9-s2-s2 (2008). 18834493 PMC2559986

[b46] Wei C. H. & Kao H. Y. Cross-species gene normalization by species inference. BMC Bioinformatics 12 **Suppl 8**, S5, doi:10.1186/1471-2105-12-S8-S5 (2011). 22151999 PMC3269940

[b47] Arighi C. N. *et al.* BioCreative III interactive task: an overview. BMC Bioinformatics 12 **Suppl 8**, S4, doi:10.1186/1471-2105-12-S8-S4 (2011). 22151968 PMC3269939

[b48] Morgan A. A. *et al.* Overview of BioCreative II gene normalization. Genome Biol. 9 **Suppl 2**, S3, doi:10.1186/gb-2008-9-s2-s3 (2008). 18834494 PMC2559987

[b49] Hakenberg J. *et al.* The GNAT library for local and remote gene mention normalization. Bioinformatics 27, 2769–2771, doi:10.1093/bioinformatics/btr455 (2011). 21813477 PMC3179658

[b50] Neves M. L., Carazo J. M. & Pascual-Montano A. Moara: a Java library for extracting and normalizing gene and protein mentions. BMC Bioinformatics 11, 157, doi:10.1186/1471-2105-11-157 (2010). 20346105 PMC2851609

[b51] Leaman R., Islamaj Dogan R. & Lu Z. DNorm: disease name normalization with pairwise learning to rank. Bioinformatics 29, 2909–2917, doi:10.1093/bioinformatics/btt474 (2013). 23969135 PMC3810844

[b52] Aronson A. R. Effective mapping of biomedical text to the UMLS Metathesaurus: the MetaMap program. Proc. AMIA Symp. , 17–21 (2001). 11825149 PMC2243666

[b53] Wei C. H., Kao H. Y. & Lu Z. PubTator: a web-based text mining tool for assisting biocuration. Nucleic Acids Res. 41, W518–522, doi:10.1093/nar/gkt441 (2013). 23703206 PMC3692066

[b54] Mattingly C. J., Colby G. T., Forrest J. N. & Boyer J. L. The Comparative Toxicogenomics Database (CTD). Environ Health Perspect. 111, 793–795 (2003). 12760826 10.1289/ehp.6028 PMC1241500

[b55] Douglas S. M., Montelione G. T. & Gerstein M. PubNet: a flexible system for visualizing literature derived networks. Genome Biol. 6, R80, doi:10.1186/gb-2005-6-9-r80 (2005). 16168087 PMC1242215

[b56] Ashburner M. *et al.* Gene ontology: tool for the unification of biology. The Gene Ontology Consortium. Nat. Genet. 25, 25–29, doi:10.1038/75556 (2000). 10802651 PMC3037419

[b57] Szklarczyk D. *et al.* The STRING database in 2011: functional interaction networks of proteins, globally integrated and scored. Nucleic Acids Res. 39, D561–568, doi:10.1093/nar/gkq973 (2011). 21045058 PMC3013807

[b58] Safran M. *et al.* GeneCards Version 3: the human gene integrator. Database (Oxford) 2010, baq020, doi:10.1093/database/baq020 (2010). 20689021 PMC2938269

[b59] Barrett T. *et al.* NCBI GEO: archive for functional genomics data sets--update. Nucleic Acids Res. 41, D991–995, doi:10.1093/nar/gks1193 (2013). 23193258 PMC3531084

[b60] Wishart D. S. *et al.* DrugBank: a knowledgebase for drugs, drug actions and drug targets. Nucleic Acids Res. 36, D901–906, doi:10.1093/nar/gkm958 (2008). 18048412 PMC2238889

[b61] Jourquin J., Duncan D., Shi Z. & Zhang B. GLAD4U: deriving and prioritizing gene lists from PubMed literature. BMC Genomics 13 **Suppl 8**, S20, doi:10.1186/1471-2164-13-S8-S20 (2012). 23282288 PMC3535723

[b62] Wren J. D. Extending the mutual information measure to rank inferred literature relationships. BMC Bioinformatics 5, 145, doi:10.1186/1471-2105-5-145 (2004). 15471547 PMC526381

[b63] Alako B. T. *et al.* CoPub Mapper: mining MEDLINE based on search term co-publication. *BMC Bioinformatics* 6, 51, doi:10.1186/1471-2105-6-51 (2005). 15760478 PMC1274248

[b64] Tsuruoka Y., Tsujii J. & Ananiadou S. FACTA: a text search engine for finding associated biomedical concepts. Bioinformatics 24, 2559–2560, doi:10.1093/bioinformatics/btn469 (2008). 18772154 PMC2572701

[b65] Rebholz-Schuhmann D. *et al.* EBIMed--text crunching to gather facts for proteins from Medline. Bioinformatics 23, e237–244, doi:10.1093/bioinformatics/btl302 (2007). 17237098

[b66] Huang D. W. *et al.* The DAVID Gene Functional Classification Tool: a novel biological module-centric algorithm to functionally analyze large gene lists. Genome Biol. 8, R183, doi:10.1186/gb-2007-8-9-r183 (2007). 17784955 PMC2375021

