# Supplementary material for: PALM-IST: Pathway Assembly from Literature Mining - an Information Search Tool
Source: Sci Rep. 2015 May 19;5:10021. doi: 10.1038/srep10021 (PMC4437304; doi:10.1038/srep10021)
Supplement: Supporting Information — Supplementary File 1 [file srep10021-s1.pdf]

Supplementary File 1

PALM-IST: Pathway Assembly from Literature Mining - an Information Search Tool.

Sapan Mandloi and Saikat Chakrabarti

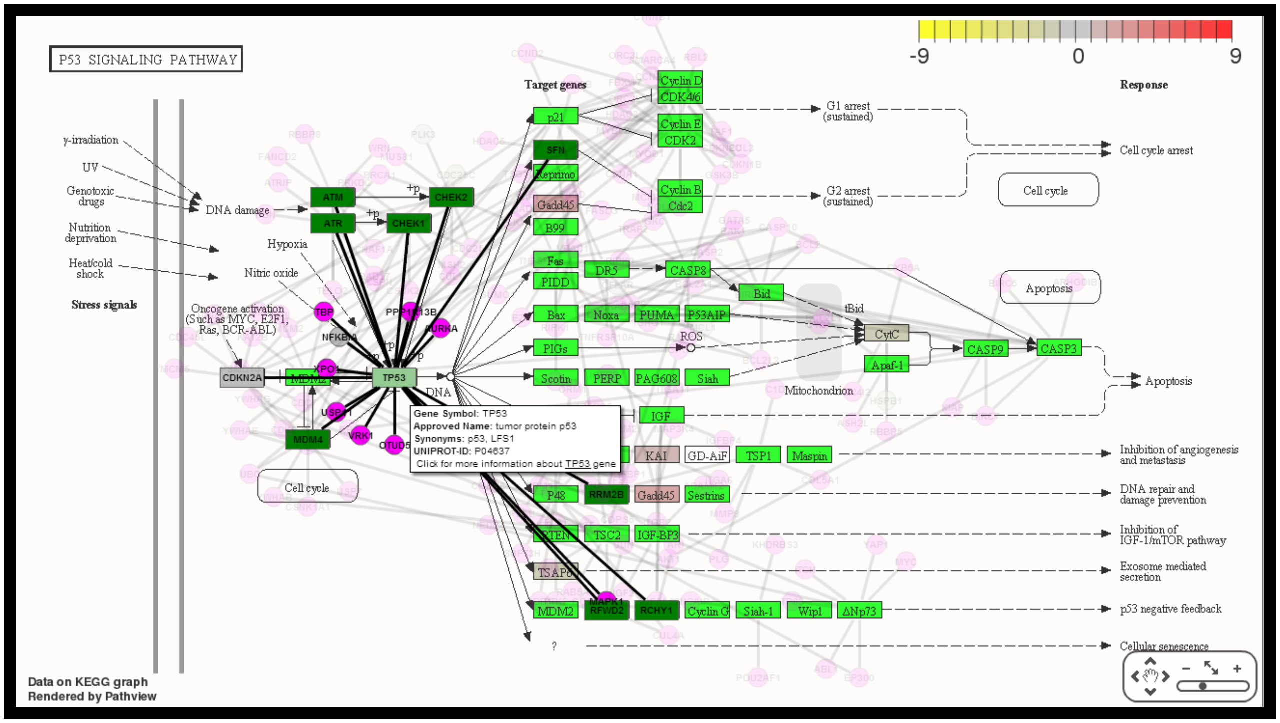

Figure S1: Expression mapped onto KEGG[1] p53 signaling pathway (copyright image of KEGG and permission to publish is obtained from Kanehisa Laboratories) overlaid with protein-protein interaction information. Differential expression is calculated for GEO series GSE16099 (effect of hypoxia on gene expression by human macrophages), filter criteria of p-value <=0.005 and log Fold Change as (logFC <= -1 or logFC >=1) is utilized. Green rectangle nodes represent non-mapped pathway genes/proteins. Differentially expressed genes/proteins in rectangle boxes are colored as yellow (with lower expression) to red (with higher expression). Circle nodes (pink: non-mapped; yellow to red: low to high expression level) represent first level protein-protein interactors of pathway proteins.

Table S1: PALM-IST statistics for multiple types of diseases as query keywords.

Query	Number of abstracts	Number of genes	Number of PPI connections of TOP10 genes	Number of pathways	Number of drugs	Crosstalk proteins	Metabolic-Signaling common proteins	TOP author
**Hypercholesterolemia(MESH : D006937)**	27667	3544	393	132	693	80	4	Kastelein JJ
**Insulin Resistance (MESH:D007333)**	56269	7500	601	125	811	71	8	Wang Y
**Diabetic Nephropathies (MESH: D003928)**	13832	2992	557	139	426	70	4	Parving HH
**METABOLIC DISEASES**								
**Hepatocellular Carcinoma, (MESH:D006528)**	46037	8014	1515	135	632	88	5	Wang Y
**Lung Neoplasms (MESH:D008175)**	76278	8860	1585	128	773	83	4	Wang Y
**Glioblastoma (MESH:D005909)**	20160	5650	1727	120	587	63	7	Weller M
**CANCER**								
**Pancreatitis (MESH:D010195)**	34472	4191	732	142	818	72	2	Büchler MW
**Endotoxemia (MESH:D019446)**	7090	2588	409	126	432	83	5	van der Poll T
**Papilloma (MESH:D010212)**	12360	2361	1155	129	323	68	2	Slaga TJ
**INFECTIOUS DISEASES**								
**Brain Ischemia (MESH: D002545)**	32543	4975	898	139	626	79	3	Wang Y
**Parkinson Disease (MESH:D010300)**	38931	4367	857	142	593	59	5	Lees AJ
**Asthma (MESH:D001249)**	65787	5287	457	121	823	81	6	Holgate ST
**OTHER DISEASES**								

TOP100 genes/proteins result from each query is used for protein-protein interaction (PPI), pathways, crosstalk proteins (involved in more than one pathway) and Signaling-Metabolic common proteins information extraction. Details of signaling-metabolic pathways and common protein list can be downloaded from
www.hpppi.iicb.res.in/ctm/downloads/all_sig_meta_common_protein.txt
www.hpppi.iicb.res.in/ctm/downloads/signaling_pathway_list.txt
www.hpppi.iicb.res.in/ctm/downloads/metabolic_pathway_list.txt

Figure S2:

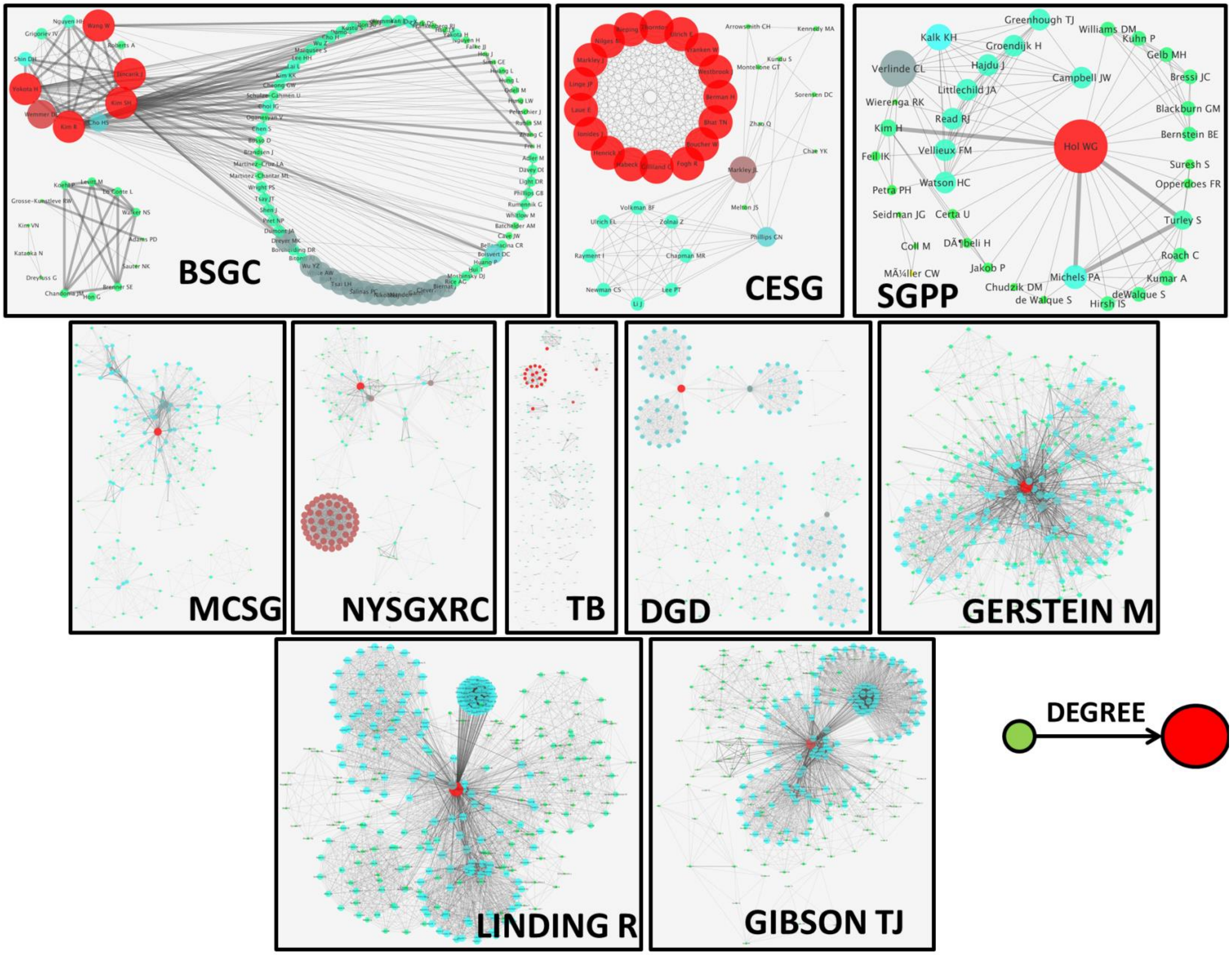

Figure S2: Input PMIDs were collected from the PubNet[2] server. Co-author networks were created from the resultant abstracts for the following research centers: Berkeley Center for Structural Genomics (BSGC), Center for Eukaroytic Structural Genomics (CESG), Structural Genomics of Pathogenic Protozoa (SGPP), Midwest Consortium for Structural Genomics (MCSG), New York Structural Genomics Research Consortium (NYSGXRC), and Tuberculosis Structural Genomics (TB) consortium. The MCSG and NYSGXRC have higher clustering and authorship of publications as individual laboratories clustering together. TB consortium has far lesser collaboration as its scientists tend to publish separately whereas the BSGC center publication created dense clusters with heavy edge weights suggesting authors participating in almost every publication. DGD (Disease Gene Drug) co-authorship network using Glioblastoma as disease, EGFR and PTEN as gene and

Erlotinib as drug keywords was created. DGD network having multiple clusters suggests that the works related to the searched bio-entities are done in multiple laboratories and ‘James CD’ is the most frequent co-author. Co-authorship network was also created for three authors: GERSTEIN M, Gibson TJ and Linding R. co-authorship networks of these authors show their extent of collaboration highlighting significant differences in magnitude and number of publication. Node (as name of the author) color and size are based on number of publication (low: green to high: red) and edge (as co-authorship connection) width are set on number of co-authored publication between the authors. Cytoscape[3] program was used to create the network figures.

Table S2: Comparison of co-authorship network properties.

Query term	Network Property									
	CC		Diameter		Density		Avg. Degree		Path Length	
	A	B	A	B	A	B	A	B	A	B
BSGC	0.895	0.909	3	3	0.095	0.095	9.245	9.093	1.966	1.966
CESG	0.939	0.972	3	3	0.295	0.329	11.805	11.2	1.932	1.374
SGPP	0.925	0.925	3	3	0.186	0.186	6.703	6.073	2.05	2.05
MCSG	0.869	0.862	4	4	0.065	0.066	10.747	10.691	2.386	2.432
NYSGXRC	0.90	0.91	4	4	0.112	0.111	20.308	19.184	2.507	2.106
TB	0.928	0.925	4	4	0.022	0.023	6.743	6.805	1.966	1.97
DGD	0.979	0.972	3	3	0.067	0.069	13.393	13.678	1.763	1.626
GERSTEIN M	0.861	0.867	2	3	0.053	0.051	16.13	15.269	1.947	1.98
LINDING R	0.94	0.939	2	2	0.071	0.078	23.146	23.18	1.929	1.922
GIBSON TJ	0.908	0.908	3	3	0.099	0.099	23.877	23.859	1.909	1.889
P-Value between A and B groups	0.78		0.75		0.91		0.92		0.38	

CC: Clustering Coefficient; A: PubNet[15] result; B: PALM-IST result

From Table S2 it is clear that the topological properties of co-author networks created using the PALM-IST and PubNet[2] servers are very similar. P-values of topological properties (like Clustering Coefficient, Network diameter, Network density, Average degree and path length) distribution calculated using the Cytoscape NetworkAnalyser[4] also suggest the similarity between two networks. The slight differences between the topological properties might arise due to difference in number of publications PALM-IST used to create the networks. PALM-IST only uses abstracts that contain articles for processing and does not consider the one without an abstract text.

Table S3: List of resources utilized within the PALM-IST server.

Resource	Full Name	Information collected	Data size	Reference
Entrez Gene	NCBI Entrez Gene	Gene information	15488736	5
TAXONOMY	NCBI Taxonomy	classification and nomenclature for all of the organisms	1105810	5
KEGG	Kyoto Encyclopedia of Genes and Genomes	Pathways in all organisms	123650	1
DrugBank	DrugBank database	Approved Drug name	1452	6
STRING	STRING Database	Protein-Protein interaction	16826 (score >=0.7)<br>77730 (score >=0.4)	7
GeneCards	GeneCards Database	Human Gene information	23184	8
GEO	Gene Expression Omnibus	High throughput Expression Series data	48283<br>(Available online)<br>(Date of access: 24/08/2014)	9
CTD	The Comparative Toxicogenomics Database	Interaction/association between bio-entities	http://ctdbase.org/about/dataStatus.go<br>(Date of access: 24/08/2014)	10
GO	Gene Ontology Database	Biological Processes	25370	11
		Cellular localization	3294	
Pubtator	Pubtator	Gene and chemical/drug annotations	NA	12
DNorm	Disease Normalization	Disease Name	NA	13
BioCreative	Critical Assessment of Information Extraction in Biology	NER Test corpus for evaluation	NA	14

Table S4: PALM-IST data size.

**Total Number of Articles**	**14361661**
**Number of articles tagged with gene/protein**	**4742754**
**Number of articles tagged with disease**	**8911109**
**Number of articles tagged with approved drug**	**2873208**
**Number of articles tagged with GO processes**	**6483693**

Gene name recognition programs

GeneTUKit:

GeneTUKit[15] is document level gene normalization software for full-text articles. This software employs both local context surrounding gene mentions and global context from the full-text document. GeneTUKit comprises of four main steps from gene name identification to gene ID generation. The first step is gene name recognition performed by ABNER[16] and conditional random field (CRF) based program. Consensus result of step one is used in step two to generate ID for gene mention. Gene ID disambiguation is performed in third step using trained ranking algorithm ListNet[17]. In the last step, support vector machine (SVM) based score is given to each ID. In PALM-IST, GeneTUKit is implemented as entity recognition tool for gene mention.

BANNER:

BANNER[18] is a NER system that identifies gene and protein symbols in text through a machine learning approach based on conditional random fields. Performance of BANNER was compared with the GeneTUKit tool embedded within the PALM-IST for gene mention.

Abgene:

Abgene[19] is a gene/protein name tagger trained on MEDLINE abstracts and uses a combination of statistical and rule-based strategies. In this study, Abgene was used for comparison for gene mention benchmarking.

Gene normalization programs

GenNorm

GenNorm[20] is an open source software tool for gene normalization in biomedical text. GenNorm associates gene mention with relevant organisms, and assigning them with corresponding NCBI Gene IDs. GenNorm achieved the best performance in the BioCreative III gene normalization challenge task[21]. GenNorm uses three modules for gene normalization task, such as gene name recognition module, species assignation module and

species-specific gene normalization module. GeneTUKit is used in PALM-IST as gene name recognition module for GenNorm. For species assignation, species mention task is performed using dictionary based lookup method and later multiple filter of disambiguation is applied. Rule based species assignation strategy was applied for final species assignation. In the last step, species-specific gene normalization module was applied to assign score for each assignment. In PALM-IST, GenNorm is implemented as entity recognition tool for gene normalization.

GNAT

GNAT[22] (Gene Name Normalization) is a library and web service, which perform name entity recognition and normalization in biomedical literature. GNAT is accessible through online service and gene name normalization was available only for three organisms [Human (NCBI Taxid: 9606), Mouse (NCBI Taxid: 10090) and Fruit fly (NCBI Taxid: 7227)]. We performed benchmarking for gene normalization task at abstract level and full text level in this study.

Moara

Moara[23] is open-source text mining program for entity tagging and normalization. Moara is available as stand-alone version. We compared its benchmarking result on BC2GN corpus with other available programs.

Disease normalization programs

DNorm

DNorm[13] is a recently published disease normalization program, which uses BANNER for disease mention and pair wise 'Learning to Rank' method for normalization. First step is disease mention performed by BANNER NER program which was trained on NCBI disease training set. The next step is to create concepts for each mention terms. In last step disambiguation and multiple filtering is applied and MESH/OMIM tag is given for normalization. In PALM-IST we have used the DNorm module for disease term identification.

MetaMap

MetaMap[24] is public resource of the NLM (National Library of Medicine) which perform natural language processing (NLP) task for identifying UMLS Meta-thesaurus concepts in biomedical text. MetaMap splits the input text into sentences and phrases to identify possible mappings to UMLS based on lexical lookup.

Chemical/Drug name recognition program

PubTator

PubTator[12] is a web-based tool that allows curators to create, save, and export annotations through the use of advanced text-mining techniques. In PALM-IST we have used the annotation of PubTator for approved drug mining task.

Whatizit

Whatizit[25] is a text mining system to identify biomedical terms from the text. Multiple pipelines are available online and in Simple Object Access Protocol (SOAP) form. In this study we have used Whatizit SOAP services to identify approved drugs from the literature abstract and benchmarking is performed.

Gene Ontology (GO) biological process name recognition program

PALM-ISTag

For identification of biological processes PALM-IST used dictionary based lookup approach where it uses 64687 Gene Ontology[11] processes. Tagged GO process is highlighted in abstract display, which can help reader to quickly relate biological process with other bio-entities. Co-occurrence of GO processes with gene/protein, drug and disease is provided in tabular and interactive network under interaction from text result section of PALM-IST.

Figure S3:

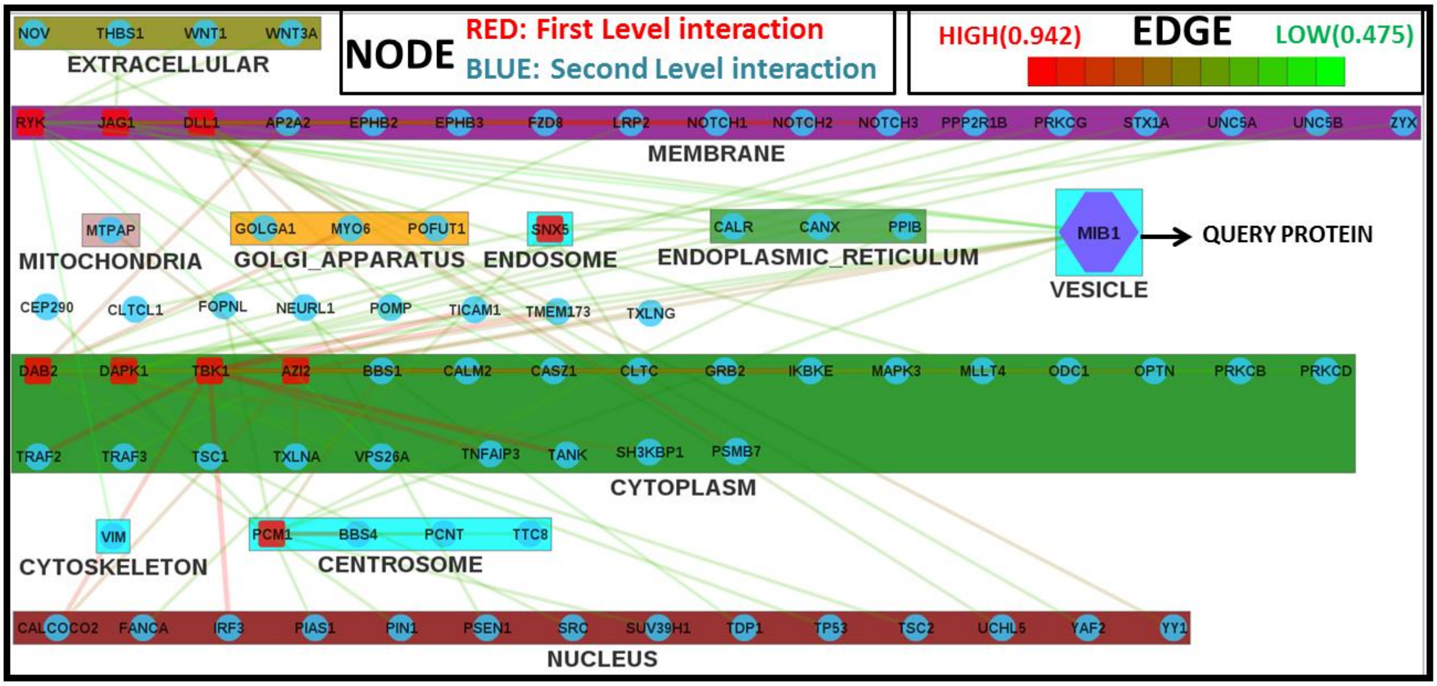

Figure S3: Protein-protein interaction of MIB1 (mindbomb E3 ubiquitin protein ligase 1) protein [present within TOP15 genes/proteins result of PALM-IST example run] localized in cytoplasmic vesicle (GO: 0031410; source: Ensembl) with STRING experimental cut-off as >=0.4. MIB1 interact with 10 proteins (in RED node). These 10 proteins make 77 interactions involving 71 proteins (in BLUE node). The interactive network with its topological properties is available at www.hpppi.iicb.res.in/ctm/interaction/MIB1.int2.html

Expression mapping onto pathways

PALM-IST provides users an option to map gene/protein expression information onto pathways. PALM-IST utilizes Pathview[26] package for pathway based data integration and visualization of molecular expression data. To compare two groups of ‘samples’ in a GEO Series[9] in order to identify genes that are differentially expressed across experimental conditions we have used GEOquery[27], R 3.1.0, Biobase[28] and LIMMA[29] packages. Pathview automatically downloads the KEGG pathway graph data, parse the data and map the expression information (Figure S4) either provided by the user or directly fetched from the corresponding GEO series. File format required for user to map expression data is provided below. GEO series based differential expression calculation is performed by downloading GSE details online. Dataset for input GSE series provided by user is downloaded and parsed into R data structure using GEOquery package. Parsed data from the GEOQuery is given as input to LIMMA (Linear Models for Microarray Analysis) package. P-value and $\log_2$ fold-change (FC) is calculated by LIMMA package based on user selected control and reference samples in GSE series. P-value cut-off of 0.005 and $\log_2$ FC of ±1 is used for identifying differentially expressed genes.

PALM-IST takes two types of input for gene expression mapping:

1. File in TAB delimited format. Column1 as Entrez GeneID and column2 as fold change value.
   Example:
   57125 -1.833338977
   7078 -5.881084239
   11118 -1.334781693
   7022 -3.8036085
   5289 -1.3952710515
   6423 -3.4752778695
   914 5.6444686505
   3575 -4.889690576
   637 -4.0863627285
   2202 -4.86902651

2. GEO series name for differential expression calculation.

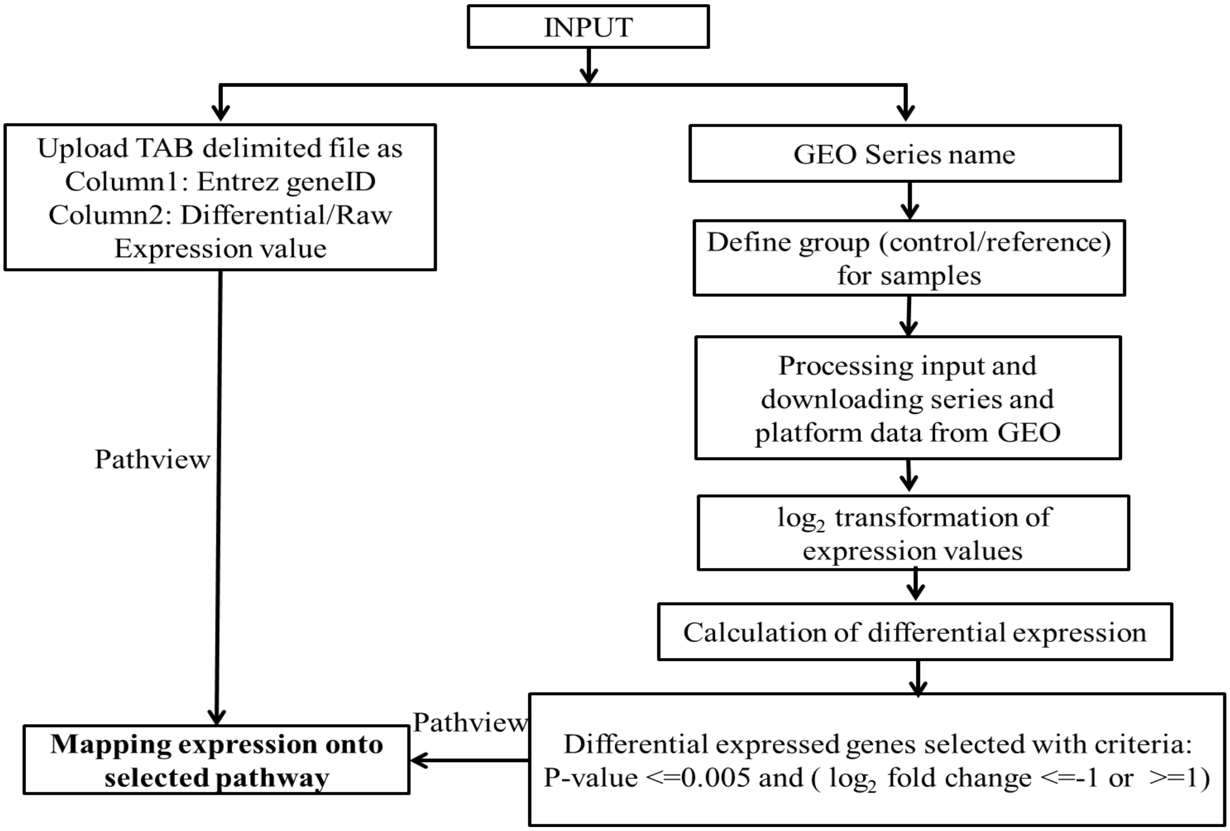

Figure S4: Complete workflow for expression mapping onto pathways and differential expression calculation.

References:

1 KEGG: kyoto encyclopedia of genes and genomes. *Nucleic Acids Res* **28**, 27-30 (2000).

2 Douglas, S. M., Montelione, G. T. & Gerstein, M. PubNet: a flexible system for visualizing literature derived networks. *Genome Biol* **6**, R80, doi:10.1186/gb-2005-6-9-r80 (2005).

3 Smoot, M. E., Ono, K., Ruscheinski, J., Wang, P. L. & Ideker, T. Cytoscape 2.8: new features for data integration and network visualization. *Bioinformatics* **27**, 431-432, doi:10.1093/bioinformatics/btq675 (2011).

4 Doncheva, N. T., Assenov, Y., Domingues, F. S. & Albrecht, M. Topological analysis and interactive visualization of biological networks and protein structures. *Nat Protoc* **7**, 670-685, doi:10.1038/nprot.2012.004 (2012).

5 Database resources of the National Center for Biotechnology Information. *Nucleic Acids Res* **42**, D7-17, doi:10.1093/nar/gkt1146 (2014).Kanehisa, M. & Goto, S.

6 Wishart, D. S. *et al.* DrugBank: a knowledgebase for drugs, drug actions and drug targets. *Nucleic Acids Res* **36**, D901-906, doi:10.1093/nar/gkm958 (2008).

7 Szklarczyk, D. *et al.* The STRING database in 2011: functional interaction networks of proteins, globally integrated and scored. *Nucleic Acids Res* **39**, D561-568, doi:10.1093/nar/gkq973 (2011).

8 Safran, M. *et al.* GeneCards Version 3: the human gene integrator. *Database (Oxford)* **2010**, baq020, doi:10.1093/database/baq020 (2010).

9 Barrett, T. *et al.* NCBI GEO: archive for functional genomics data sets--update. *Nucleic Acids Res* **41**, D991-995, doi:10.1093/nar/gks1193 (2013).

10 Mattingly, C. J., Colby, G. T., Forrest, J. N. & Boyer, J. L. The Comparative Toxicogenomics Database (CTD). *Environ Health Perspect* **111**, 793-795 (2003).

11 Ashburner, M. *et al.* Gene ontology: tool for the unification of biology. The Gene Ontology Consortium. *Nat Genet* **25**, 25-29, doi:10.1038/75556 (2000).

12 Wei, C. H., Kao, H. Y. & Lu, Z. PubTator: a web-based text mining tool for assisting biocuration. *Nucleic Acids Res* **41**, W518-522, doi:10.1093/nar/gkt441 (2013).

13 Leaman, R., Islamaj Dogan, R. & Lu, Z. DNorm: disease name normalization with pairwise learning to rank. *Bioinformatics* **29**, 2909-2917, doi:10.1093/bioinformatics/btt474 (2013).

14 Blaschke, C., Hirschman, L., Yeh, A. & Valencia, A. Critical assessment of information extraction systems in biology. *Comp Funct Genomics* **4**, 674-677, doi:10.1002/cfg.337 (2003).

15 Huang, M., Liu, J. & Zhu, X. GeneTUKit: a software for document-level gene normalization. *Bioinformatics* **27**, 1032-1033, doi:10.1093/bioinformatics/btr042 (2011).

16 Settles, B. ABNER: an open source tool for automatically tagging genes, proteins and other entity names in text. *Bioinformatics* **21**, 3191-3192, doi:10.1093/bioinformatics/bti475 (2005).

17 Cao, Z. *et al*. Learning to rank: from pairwise approach to listwise approach. *Proceedings of the 24th International Conference on Machine Learning*, doi:10.1145/1273496.1273513 (2007).

18 Leaman, R. & Gonzalez, G. BANNER: an executable survey of advances in biomedical named entity recognition. *Pac Symp Biocomput*, 652-663, doi:10.1142/9789812776136_0062 (2008).

19 Tanabe, L. & Wilbur, W. J. Tagging gene and protein names in biomedical text. *Bioinformatics* **18**, 1124-1132 (2002).

20 Wei, C. H. & Kao, H. Y. Cross-species gene normalization by species inference. *BMC Bioinformatics* **12 Suppl 8**, S5, doi:10.1186/1471-2105-12-S8-S5 (2011).

21 Lu, Z. *et al.* The gene normalization task in BioCreative III. *BMC Bioinformatics* **12 Suppl 8**, S2, doi:10.1186/1471-2105-12-S8-S2 (2011).

22 Hakenberg, J. *et al.* The GNAT library for local and remote gene mention normalization. *Bioinformatics* **27**, 2769-2771, doi:10.1093/bioinformatics/btr455 (2011).

23 Neves, M. L., Carazo, J. M. & Pascual-Montano, A. Moara: a Java library for extracting and normalizing gene and protein mentions. *BMC Bioinformatics* **11**, 157, doi:10.1186/1471-2105-11-157 (2010).

24 Aronson, A. R. Effective mapping of biomedical text to the UMLS Metathesaurus: the MetaMap program. *Proc AMIA Symp*, 17-21 (2001).

25 Rebholz-Schuhmann, D., Arregui, M., Gaudan, S., Kirsch, H. & Jimeno, A. Text processing through Web services: calling Whatizit. *Bioinformatics* **24**, 296-298, doi:10.1093/bioinformatics/btm557 (2008).

26 Luo, W. & Brouwer, C. Pathview: an R/Bioconductor package for pathway-based data integration and visualization. *Bioinformatics* **29**, 1830-1831, doi:10.1093/bioinformatics/btt285 (2013).

27 Davis, S. & Meltzer, P. S. GEOquery: a bridge between the Gene Expression Omnibus (GEO) and BioConductor. *Bioinformatics* **23**, 1846-1847, doi:10.1093/bioinformatics/btm254 (2007).

28 Gentleman, R. C. *et al.* Bioconductor: open software development for computational biology and bioinformatics. *Genome Biol* **5**, R80, doi:10.1186/gb-2004-5-10-r80 (2004).

29 Smyth, G.K. Limma: linear models for microarray data. Bioinformatics and Computational Biology Solutions Using {R} and Bioconductor, 397-420, doi:10.1007/0-387-29362-0_23 (2005).
